# Neural Stem Cell Plasticity: Advantages in Therapy for the Injured Central Nervous System

**DOI:** 10.3389/fcell.2017.00052

**Published:** 2017-05-12

**Authors:** Linda Ottoboni, Arianna Merlini, Gianvito Martino

**Affiliations:** Neuroimmunology Unit, Division of Neuroscience, Institute of Experimental Neurology, San Raffaele Scientific InstituteMilan, Italy

**Keywords:** neural stem cells, microenvironment, plasticity, metabolism, inflammation, stroke, multiple sclerosis, aging

## Abstract

The physiological and pathological properties of the neural germinal stem cell niche have been well-studied in the past 30 years, mainly in animals and within given limits in humans, and knowledge is available for the cyto-architectonic structure, the cellular components, the timing of development and the energetic maintenance of the niche, as well as for the therapeutic potential and the cross talk between neural and immune cells. In recent years we have gained detailed understanding of the potentiality of neural stem cells (NSCs), although we are only beginning to understand their molecular, metabolic, and epigenetic profile in physiopathology and, further, more can be invested to measure quantitatively the activity of those cells, to model *in vitro* their therapeutic responses or to predict interactions *in silico*. Information in this direction has been put forward for other organs but is still limited in the complex and very less accessible context of the brain. A comprehensive understanding of the behavior of endogenous NSCs will help to tune or model them toward a desired response in order to treat complex neurodegenerative diseases. NSCs have the ability to modulate multiple cellular functions and exploiting their plasticity might make them into potent and versatile cellular drugs.

## Introduction

Although it has been thought for a long time that mammalian neurogenesis occurs only during embryonic and perinatal stages, young neurons are continuously incorporated into the adult brain as demonstrated by Altman and Das already in the early sixties (Altman, 1962; Altman and Das, 1965). Indeed, neural stem cells (NSCs), residing in the brain of most adult mammals in the so-called “neurogenic niches,” sustain neurogenesis throughout life. It is estimated that 700 new neurons are generated every day by the neuropoietic niche in an adult human hippocampus, outlining one aspect of the plasticity/renewal capacity of NSCs (Knoth et al., 2010; Spalding et al., 2013). As for other stem cells, the specialized microenvironment of the neurogenic niche ensures not only NSC self-renewal but also differentiation, mainly into neurons. However, neurogenesis is not the only activity of NSCs in the adulthood. As a matter of fact, recent studies indicate that adult NSCs residing within the sub-ventricular zone (SVZ) might physiologically exert alternative functions to cell replacement, the so-called non-neurogenic functions (Martino et al., 2014), mainly aimed at protecting CNS homeostasis, in both physiological and pathological conditions. They regulate and are regulated by several signaling pathways (Faigle and Song, 2013) that, tuning the evolution of progenitor proliferation, division, and migration, can *per se* also impact the composition of the niche (Preston and Sherman, 2011; Gattazzo et al., 2014). Neighboring cells, the vasculature and the cerebrospinal fluid constitute the main routes through which molecular signals reach NSCs and affect their behavior.

Overall, knowing the physiological properties of NSCs and what changes in pathological conditions opens up the possibility of exploiting NSC plasticity for preventive/therapeutic purposes.

This review will primarily focus on (i) the properties of precursors of the adult neurogenic niches of the central nervous system (CNS); (ii) the mechanisms of inter- and intra-cellular communication of NSCs and other cells, resident or not in the niche, in physio- and patho-logical conditions, with focus on multiple sclerosis (MS) and ischemic stroke, neurodegenerative disorders of the brain that unfold acute and chronic consequences.

## What defines a NSC and a NSC niche?

At the onset of murine neurogenesis, at embryonic day 9.5, the precursors in the CNS are neuroepithelial cells (NECs) that form a tube with a central canal (Taverna et al., 2014). NECs are highly proliferative and initially divide symmetrically to expand; afterwards they convert into radial glial cells (RGCs) that divide both symmetrically and asymmetrically. Basal processes of RGCs are used by newborn neurons as guiding scaffolds while they migrate away from the germinal niche toward the pial surface.

Although most CNS regions largely extinguish their NSC pool after development, discrete areas of the adult brain retain NSCs and active neurogenesis throughout life (Ming and Song, 2005, 2011). Namely, the striatal subventricular zone (SVZ) and the hippocampal dentate gyrus (DG, subgranular zone SGZ) are the most extensively characterized adult neurogenic niches. However, according to the most recent evidences, sites of neurogenesis are present also in the ependyma (Alvarez-Buylla and Lim, 2004; Bjornsson et al., 2015), near the third and fourth ventricle, in the forebrain, in the striatum, in the amygdala, in the hypothalamus, in the substantia nigra and in the subcortical white matter or spinal cord root ganglia (Bernier et al., 2002; Lie et al., 2002; Kokoeva et al., 2005; Chang et al., 2008; Ernst et al., 2014; Muratori et al., 2015; Stolp and Molnar, 2015). Proliferating cells from those regions, namely somatic NSCs, can be isolated and established as virtually perpetual cell lines in response to fibroblast growth factor 2 (FGF-2) and epidermal growth factor (EGF) similar to their embryonic counterparts (Temple, 2001).

In the adult neural stem cell niche, NSCs, immature neurons, supporting astrocytes, blood vessels and epithelial ciliated cells are in close contact and the vasculature with “leaky” features supports adult neurogenesis (Butti et al., 2014). In the mouse, the SVZ contains slowly dividing progenitors that can be subdivided into two types: type B1 cells, in close contact with both the cerebrospinal fluid (CSF) and the blood vessels of the SVZ, and type B2 cells, closer to the striatum (Ihrie et al., 2011). B1 cells give rise to transit amplifying cells (type C cells), located in close proximity to blood vessels, and along with B2 cells, they form a glial supportive sheath around their more differentiated progeny and migrating neuroblasts, type A cells, that originate from type C cells. Type A cells migrate tangentially to form the rostral migratory stream (RMS) to the olfactory bulb for terminal differentiation. Once in the olfactory bulb, the neuroblasts defasciculate from the stream and migrate radially to their site of terminal differentiation into neurons (Alvarez-Buylla et al., 2000). SVZ-NSCs give rise also to oligodendrocyte precursors and mature oligodendrocytes, continuously replenishing cells in the corpus callosum (Menn et al., 2006).

The primary role of the neurogenic SGZ niche instead is to generate new granule cells, primary excitatory neurons that support hippocampus-dependent cognitive functions (Zhao et al., 2008). Stem cells of the SGZ give rise to radial astrocytes that convert into immature progenitors (Type 1, the counterpart of type B in the SVZ) and eventually into neuroblasts (Type 2, the counterpart of Type C-A cells in the SVZ) (Zhao et al., 2008). Complete depletion either of type 2 or type C cells, respectively in the SGZ and SVZ, (non-radial glia like cells) stops neurogenesis (Doetsch et al., 1999; Ahn and Joyner, 2005), although, but infrequently, dividing radial glia could sustain neurogenesis as well (Seri et al., 2004). NSCs of the SGZ are also in close contact with blood vessels and endothelial cells, that act as scaffolding cells for NSCs (Palmer et al., 2000) and play a major role in directing NSC specification (Shen et al., 2004, 2008; Tavazoie et al., 2008; Kokovay et al., 2010).

This is the conventional cell classification for the mouse neural germinal center, which has been better characterized.

In humans the SVZ differs from the one in rodents because it organizes into four layers instead: the ependymal layer, the hypocellular gap, the astrocytic ribbon, and the transitional zone to the parenchyma, rich in myelin and oligodendrocytes (Quinones-Hinojosa et al., 2006). Migrating neuron-like cells can occasionally be found in the layers II and III as individual cells (Sanai et al., 2004; Quinones-Hinojosa et al., 2006). In detail, layer I consists of an ependymal monolayer lining the ventricular wall with astrocytic processes contacting the ventricular wall. Layer II, also known as the gap region, is rich in GFAP^+^ processes, with only some neuroblasts in the anterior regions. Ependymal cells send basal processes into Layer II, making critical contacts with the underlying basal lamina. Layer II may function as the corridor for neuroblast migration. Layer III is the proliferative region of the human SVZ, with GFAP^+^/Ki67^+^ and CD133^+^ cells. Few neuroblasts are present in the human SVZ, compared to the rodent, and are found mainly in Layer III. Some ependymal cells, which typically comprise the epithelial barrier of the ventricles, have motile cilia and are found in small clusters (4–14 cells) in Layer III (Quinones-Hinojosa et al., 2006; Kam et al., 2009). Layer IV represents the first portion of brain parenchyma away from the parietal ventricle and where the first evidence of neurons is found.

## Extracellular cues and intrinsic genetic programs control the NSC functions in physiological conditions in the adult brain

**Figure 1:**
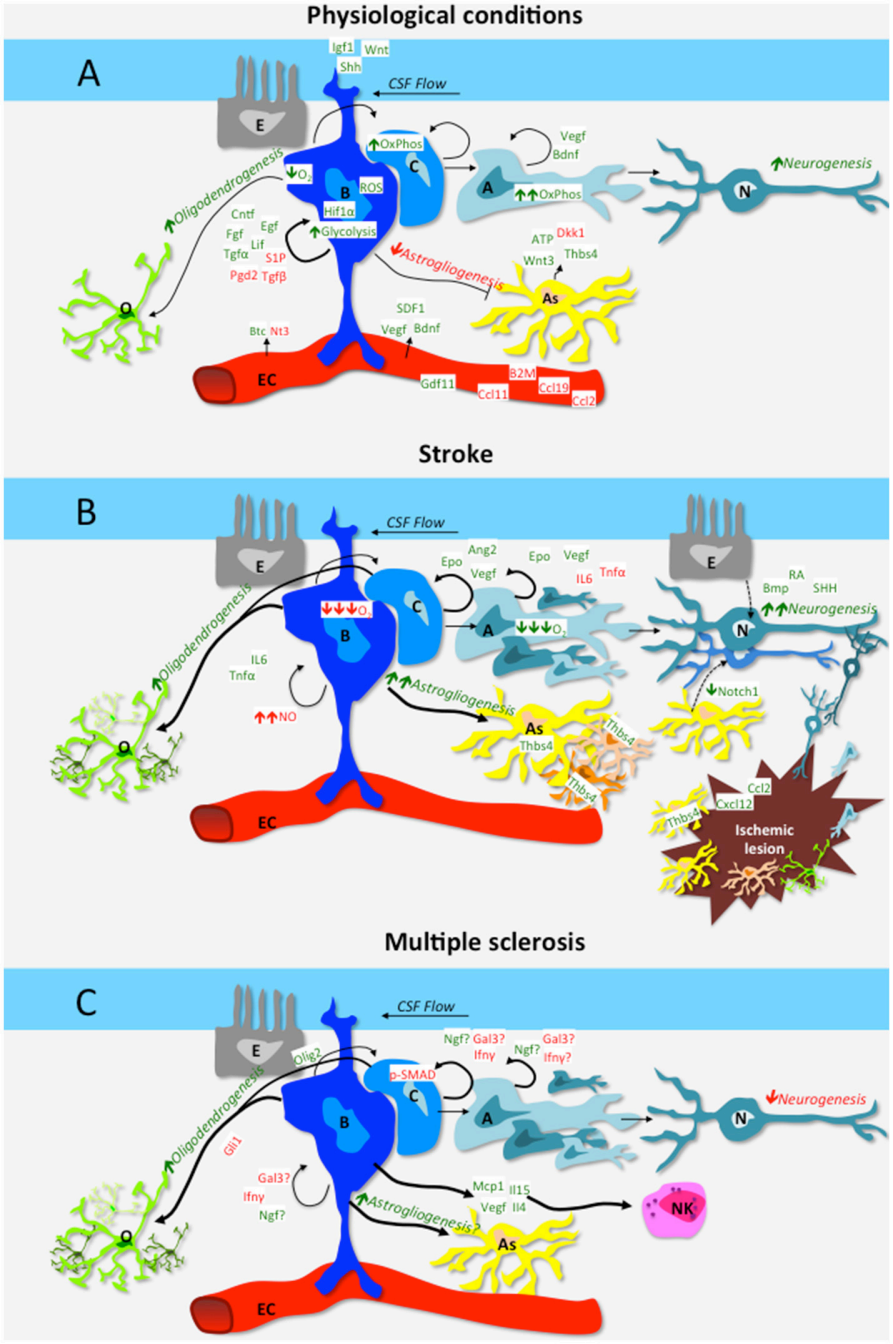
**Schematic representation of the interplay among cells of the neural stem cell niche of the subventricular zone (A,B,C)**, vascular endothelial cells (EC), ependymal cells (E), differentiated oligodendrocytes (O), astrocytes (As), and neurons (N). Green is used for positive regulators of neural stem cell function, red for inhibitory regulators. Mechanisms are in italic. **(A)** depicts mechanisms and factors in physiological conditions: in steady-state, B cells self renewal is promoted by niche-derived factors such as CNTF, EGF, FGF2, LIF, PGD2, S1P, and TGFα, as well as by systemic-derived factors as VEGF, BDNF and SDF1. The cerebrospinal fluid also contributes actively to niche homeostasis *via* IGF1, Wnt and Shh that signal to B cells *via* their apical cilium. Aging increases neurogenesis-inhibitory factors such as B2M, CCL2, CCL11, CCL19, while pro-neurogeneic factors as GDF11 decrease. The hypoxic milieu of the niche favors B cell quiescence, while C and A precursors rely on oxidative phosphorylation. In steady-state, astrogliogenesis is generally inhibited, while growing astrocytes secrete both pro-neurogeneic and anti-neurogeneic mediators. Nonetheless, a basal level of oligodendrogenesis and in particular neurogenesis occurs also during the steady state. **(B)** depicts mechanisms and factors that are altered in the SVZ niche in the context of stroke. Ischemia increases Epo, Ang2 and VEGF as well as morphogens BMP, RA and SHH, which stimulate neurogenesis. Moreover, chemotactic and growth factors produced within the lesion (e.g. CXCL12, CCL2) guide newly formed glial and neuronal cells toward the ischemic area. Hypoxia and increased nitric oxide inhibit B cell cycling while low O_2_ promotes precursor differentiation. Direct transdifferentiation (dashed arrow lines) from ependymal cells and astrocytes to neurons might also contribute to stroke-induced neurogenesis. Stroke *per se* increases oligodendrogenesis and astrogliogenesis as well. In particular, SVZ-derived, Thbs4 positive astrocytes are pivotal in containing tissue damage and preventing hemorrhagic transformation. **(C)** depicts mechanisms and factors that are altered in the SVZ niche in the context of MS. Neurogenesis in inhibited by IFNγ, Gal3 and upregulation of phoshorylated-SMAD (pSMAD) in neurogenic precursors. IFNγ also inhibits oligodendrogenesis *via* upregulation of Gli1. NSCs produce a wide array of soluble mediators, including IL15 that attract NK cells, which in turn contribute to the neurogenic niche dysfunction observed in MS models.

Main feature of NSCs is their plasticity (Suh et al., 2009; Martino et al., 2011). The two major adult stem neurogenic niches take advantage of different mechanisms to exploit this property, which manifests as self renewal capacity, quiescence, metabolic modulation, homing, differentiation capacity, cellular cross-talk, and immune surveillance (Figure 1A, Table 1).

**Table 1:** **Evidences from the literature are schematically reported**.

**Molecule**	**Physio-pathology**	**Source**	**Outcome**	**References**
Acetylcholine	Physiology	(ChAT) (+) neurons	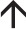 neurogenesis (synergizing with FGF2)	Paez-Gonzalez et al., 2014
Angiopoietin 2	Stroke	SVZ neuroblasts, endothelial cells	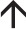 NSC and neuroblast migration 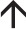 NSC neurogenic differentiation	Cui et al., 2009; Liu et al., 2009
ANKYRIN3	Physiology	Ependymal cells	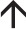 neuroblasts	Paez-Gonzalez et al., 2011
ATP	Physiology	Astrocytes	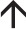 NSC proliferation	Cao et al., 2013
B2M	Aging (increases)	Blood	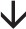 neurogenesis	Smith L. K. et al., 2015
BDNF	Physiology and stroke		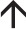 NSC differentiation	Chen et al., 2005
Betacellulin (BTC)	Physiology	Endothelial cells	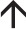 NSC proliferation	Gomez-Gaviro et al., 2012
BMP4	Physiology	Ependymal cells	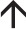 glial differentiation	Gajera et al., 2010
CCL11	Aging	Blood	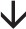 neurogenesis	Villeda et al., 2011
CNTF	Steady-state	A subtype of B cells of the SVZ, other?	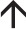 NSC self-renewal 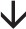 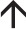 NSC neurogeneic differentiation	Emsley and Hagg, 2003; Lee et al., 2013
Decorin	Steady-state	Astrocytes	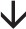 NSC neurogeneic differentiation	Barkho et al., 2006
Delta-like-4	Steady-state	Endothelial cells	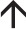 NSC proliferation	Androutsellis-Theotokis et al., 2010
Dickkopf-1	Aging	NSCs	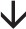 neurogenesis	Seib et al., 2013
Dopamine	Physiology	Dopaminiergic neurons	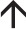 neurogenesis (synergizing with with EGF)	O'Keeffe et al., 2009
Ephrin-B2	Steady-state	Astrocyte	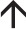 NSC proliferation 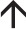 neurogenesis	Ashton et al., 2012
EPO	Hypoxia, stroke	Endothelial cells, Blood	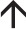 NSC proliferation and survival	Pavlica et al., 2012
FGF2	Physiology	Astrocytes	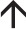 NSC proliferation and survival	Shetty et al., 2005; Widera et al., 2006
GABA	Physiology	Young neuroblasts	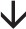 NSC proliferation and neuronal differentiation	Liu et al., 2005
GDF11	Aging (decreases)	Blood	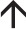 neurogenesis	Katsimpardi et al., 2014
Galectin-3	MS	SVZ	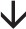 NSC proliferation	James et al., 2016
Glutamate	Physiology	Tissue	neuroblast survival	Platel et al., 2010
Gonadotropin- releasing hormone (GrH)	Aging	Hypothalamic cells	proliferating activity of hypothalamic NPC	Zhang et al., 2013
IFNγ	Stroke, MS, steady-state	Immune cells, NSCs	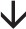 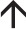 NSC proliferation 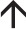 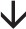 Differentiation	Pluchino et al., 2008; Li et al., 2010b; Kulkarni et al., 2016
IGF1	Steady-state, aging	Microglia, endothelial cells	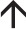 NSC proliferation 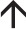 NSC neuronal differentiation 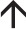 glial development	Butovsky et al., 2006; Joseph D'Ercole and Ye, 2008; Llorens-Martin et al., 2009
IGF2	Steady-state, CNS tumor, development, aging	Cerebrospinal fluid	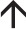 NSC proliferation	Lehtinen and Walsh, 2011
IGFBP6	Steady-state	Astrocytes	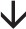 neurogenic differentiation	Barkho et al., 2006
IL10	Steady-state, stroke	T_reg_	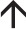 NSC proliferation 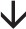 neurogenic differentiation	Perez-Asensio et al., 2013; Wang et al., 2015
IL1β	Stroke, MS	Microglia, NSCs, monocyte/macrophages?	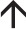 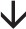 NSC proliferation 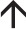 NSC apoptosis 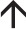 gliogenic differentiation	Wu et al., 2013 Widera et al., 2006; Guadagno et al., 2015
IL6	Infections, stroke	NSCs, microglia	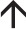 NSC proliferation 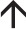 neuroblast survival	Gallagher et al., 2013; Chucair-Elliott et al., 2014; Meng et al., 2015
Jagged1	Steady-state, MS	Astrocytes	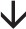 neurogenesis differentiation 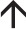 OPC proliferation	Stidworthy et al., 2004; Wilhelmsson et al., 2012
LIF	Steady-state	?	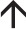 NSC proliferation	Bonaguidi et al., 2005
MCP-1/CCL2	Stroke, aging, epilepsy, CNS tumors	Immune cells? Microglia? Astrocytes?	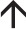 NSC migration 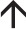 neuronal differentiation and neuritic formation of mesencephalic NSCs 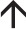 glial differentiation of NT2 NSCs	Vrotsos et al., 2009; Colucci-D'Amato et al., 2015; Osman et al., 2016
Neuregulin 1 and 2	Steady-state	Neuroblasts, GFAP+ NSCs in the SVZ	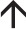 NSC proliferation 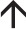 neuroblast migration	Ghashghaei et al., 2006
Neurotrophin 3 (NT3)	Steady-state	Endothelial cells	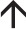 NSC quiescence 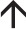 neurogenic differentiation	Shimazu et al., 2006; Delgado et al., 2014
NGF	Steady-state, MS, stroke?	SVZ	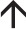 NSC proliferation 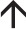 neurogenic differentiation	Calza et al., 1998; Triaca et al., 2005
Noggin	Steady-state	Ependymal cells, subgranular zone	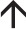 NSC proliferation 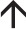 neurogenesis	Lim et al., 2000; Bonaguidi et al., 2008
Oxygen (2-5%)	Steady-state, stroke	NSCs	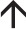 NSC self renewal 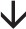 NSC differentiation	Santilli et al., 2010
Oxygen (<1%)	Stroke, MS	NSCs	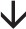 NSC self renewal 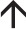 NSC differentiation	Felfly et al., 2011
PDGF	Physiology	GFAP-positive cells	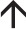 NSC proliferation	Jackson et al., 2006
PGD2	Steady-state	?	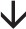 NSC proliferation	Codega et al., 2014
Retinoic acid (RA)	Stroke, steady -state	Meninges, other?	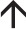 neurogenesis	Plane et al., 2008; Siegenthaler et al., 2009
ROS	Steady-state, stroke?	NSCs	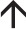 NSC self renewal 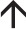 neurogenesis	Le Belle et al., 2011
S1P	Steady-state	?	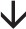 NSC proliferation	Codega et al., 2014
SDF-1/CXCL12	Stroke, MS, steady-state, traumatic brain injury	NSCs, meninges, endothelial cells, immune cells, tumor cells	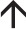 NSC migration 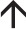 NSC survival 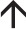 NSC differentiation	Reiss et al., 2002; Imitola et al., 2004; Itoh et al., 2009; Carbajal et al., 2010; Li et al., 2012
Serotonin	Physiology	5-HT neurons	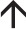 neurogenesis	Brezun and Daszuta, 1999
SHH	Development, steady-state, MS	Ventral forebrain neurons	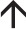 NSC specification 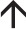 neurogenesis OPC differentiation	Breunig et al., 2008; Ihrie et al., 2011; Samanta et al., 2015
Surivivin	Aging	Astrocytes	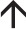 neurogenesis	Miranda et al., 2012
TGFα	Steady-state, stroke	NSCs?	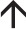 neurogenesis	Tropepe et al., 1997; Guerra-Crespo et al., 2009
TGFβ	Steady-state, development	NSCs	Temporal regulation of neurogenesis and potency of NSCs	Dias et al., 2014
Thbs4	Stroke	SVZ NSCs	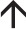 SVZ-NSC astrogenesis (via Notch signaling) 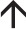 glial scar formation	Benner et al., 2013
TNFα	Stroke, MS?	Microglia, astrocytes, monocyte/macrophages?	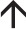 NSC proliferation 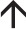 NSC apoptosis 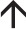 Gliogenic differentiation	Widera et al., 2006; Guadagno et al., 2013
TSP1	Steady-state	Astrocytes	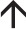 NSC proliferation 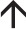 neurogenesis	Lu and Kipnis, 2010
VEGF	Steady-state, Stroke	NSCs; astrocytes; endothelial cells	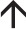 NSC proliferation and maintenance 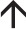 neurogenic differentiation 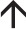 NSC migration	Kojima et al., 2010; Kirby et al., 2015
Wnt3	Steady-state	Astrocytes	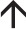 neurogenesis	Okamoto et al., 2011

The maintenance of the neurogenic niche itself and the renewal capacity depends on active intrinsic genetic (Tirone et al., 2013) and epigenetic (Liu et al., 2010; Yao et al., 2016) programs along with microenvironment-dependent specific properties. Autocrine regulators of NSC proliferation, such as transforming growth factor α (Tropepe et al., 1997; Guerra-Crespo et al., 2009) and β (Dias et al., 2014), amphyregulin, fibroblast growth factor-2 (FGF-2), insulin-like growth factor 2 (IGF2) (Marques et al., 2011) are released from specific subsets of NSCs along with leukemia inhibitory factor (LIF), ciliary neurotrophic factor (CNTF) that promote proliferation (Emsley and Hagg, 2003; Lee et al., 2013) and sphingosine-1-phosphate (S1P) or prostaglandin D2 (PGD2) that instead maintain quiescence (Codega et al., 2014; Chaker et al., 2016). The environment-contribution to NSCs plasticity encompasses several elements as described below.

Close anatomical association between NSCs and the vascular structure (vascular niche) is preserved both in the SVZ and SGZ and vascular endothelial cells of the niche secrete Notch ligands Jagged1, Jagged2, and Delta-like-4, crucial factors for self-renewal and neurogenesis (Shen et al., 2004; Androutsellis-Theotokis et al., 2010; Lu et al., 2011). Recent evidence supports the hypothesis that the cross-talk between blood vessels and NSCs is bi-directional: NSCs can indeed provide juxtacrine and paracrine signals to drive endothelial cells (Chou and Modo, 2016) and promote angiogenesis (Hicks et al., 2013). Moreover, vascular endothelial growth factor (VEGF) is a shared cue both for angiogenesis and neurogenesis because on one side it promotes the angiogenic development of capillaries, on the other, the secretion of neurogenic molecules by proximal endothelial cells (Jin et al., 2002; Cao et al., 2004; Kim et al., 2004; Udo et al., 2008; Ruiz de Almodovar et al., 2009). Further, other cues secreted from vascular endothelial cells such as neurotrophin 3 (NT3) or betacellulin (BTC) maintain quiescence or promote proliferation of NSCs, respectively (Gomez-Gaviro et al., 2012; Delgado et al., 2014), although there is evidence of NT3 effect on differentiation (Shimazu et al., 2006), while stromal derived factor-1 (SDF1) stimulates the motility of type A, B, and C neuroblasts (Kokovay et al., 2010).

The extracellular matrix (ECM), a dynamic and complex environmental element characterized by biophysical and biochemical properties specific for each tissue and able to regulate cell behavior, represents also an essential player in stem cell niche (Gattazzo et al., 2014). Extensions of the extracellular matrix known as fractones project from the blood vessels of the subventricular plexus as thin, highly branching ECM stalks that expand into bulbs where they contact the basal surface of the ependymal layer (Mercier et al., 2002). Fractones are enriched in laminin, heparan sulfate, perlecan, nidogen, and collagens. Those associations are able to bind several growth factors, suggesting that they may play a role in concentrating, activating, and presenting trophic factors to cells within the niche (Kerever et al., 2007).

NSCs receive inputs also from other cells such as microglia which reside in close proximity to NSCs of the niche. Indeed, resting microglia secrete factors that promote NSC niche maintenance and, at the same time, astrocyte differentiation of striatal NSCs *via* the Jak/Stat3 pathway (Zhu et al., 2008). Moreover, microglia contribute to the development of cytoarchitectonic and functional differences across cortical areas of the brain, secreting growth factors, and cytokines that tightly regulate the neurogenic process (Kim and de Vellis, 2005; Harry, 2013; Su et al., 2014). Conversely, *in vitro*, microglia promote neuronal differentiation, but not maintenance or self-renewal (Walton et al., 2006). On the other side, activated microglia inhibit neurogenesis (Sierra et al., 2014) favoring gliogenesis *via* tumor necrosis factor-α (TNFα) (Carpentier and Palmer, 2009), and when exposed to interleukin 4 (IL4) and interferon-γ **(**IFNγ**)**, they secrete insulin-like growth factor 1 (IGF1) and promote neuronal differentiation of NSCs (Butovsky et al., 2006). Conversely, NSCs can also influence microglia *via* VEGF that in turn modulates microglial activation, proliferation and phagocytosis (Mosher et al., 2012).

Stem plasticity is modulated by other cell types as well, namely astrocytes, residing in close proximity with NSCs both in the SVZ and in the SGZ. Their contribution to NSC proliferation is likely exploited *via* ATP release (Cao et al., 2013) while Wnt3, neurogenesin-1 (NG1), thrombospondin-1 (TSP1) as well as interleukin-1β (IL1β) and interleukin-6 (IL6) promote hippocampal neurodifferentiation (Ueki et al., 2003; Lie et al., 2005; Barkho et al., 2006; Lu and Kipnis, 2010). Of note, when FGF2-producing astrocytes age, neurogenesis is impaired (Shetty et al., 2005).

Signals arising from the ependymal and meningeal cells and released in the CSF may also influence NSC activity (Lim et al., 2000; Siegenthaler et al., 2009). Indeed, NSCs possess primary cilia which sense liquor morphogens, such as FGF2, IGF2 (effective at lower level postnatally), Wnt and Sonic Hedgehog (SHH) (Corbit et al., 2005; Rohatgi et al., 2007; Breunig et al., 2008; Kim et al., 2010; Ihrie et al., 2011; Lehtinen and Walsh, 2011) and, possibly, the CSF flow itself. The latter indeed can, *via* mechano-sensing signaling, promote proliferation and differentiation (Li et al., 2011; Arulmoli et al., 2015; Jagielska et al., 2017).

Moreover, cytoarchitectonic innervation *via* GABA (γ-Aminobutyric acid)-, glutamin-, colin-, serotonin-, and dopamin-ergic neurons sustains neurogenesis in the niche (Suh et al., 2009; Song et al., 2012; Paez-Gonzalez et al., 2014; Young et al., 2014; Alunni and Bally-Cuif, 2016; Chaker et al., 2016). Conversely, it is not clear yet whether NSCs have an impact on axons and neuronal circuitry (Zhang Y. et al., 2016).

The niche is also very much dependent on and prompt to metabolic changes. While lipid metabolism maintains proliferation and neurogenesis (structural and energy support), glycolysis regulates NSCs development and differentiation (Knobloch et al., 2013). The metabolic activity strongly depends on oxidative saturation because in mammalian CNS oxygen regulates the growth and differentiation state of stem cells (De Filippis and Delia, 2011; Ivanovic and Vlaski-Lafarge, 2016; Sandvig et al., 2017). Dividing progenitor cells depend more on glycolysis, whereas differentiated progeny relies on energetically efficient oxidative phosphorylation occurring at low oxygen concentration. Apart from those, other important metabolic pathways are active in neural stem cells, such as (i) glycogen synthesis or glutamine/folate metabolism (Goodman and Hajihosseini, 2015); (ii) phosphatidylinositol 3-kinase/AKT (PI3K/AKT) growth factor pathway insulin-dependent; (iii) mTOR pathway nutrient-dependent (Rafalski et al., 2012); (iv) AMP-activated protein kinase (AMPK)/LKB1 pathway, sensor of intracellular adenosine monophosphate (AMP) to ATP ratios, and (v) the sirtuin pathway, metabolic sensors of NAD (nicotinamide adenine dinucleotide) level and epigenetic repressors (Folmes et al., 2012; Shyh-Chang et al., 2013).

Cell to cell contact has also been shown to play a role in exploiting the plasticity of NSCs. Astrocytes of the niche negatively control neuronal differentiation through astrocyte-secreted factors such as insulin like growth factor binding protein 6 (IGFBP6) and decorin (Barkho et al., 2006; Wilhelmsson et al., 2012), while astrocytic ephrin-B2 positively regulates proliferation (Ashton et al., 2012).

Another peculiar property of NSCs consists in their capacity to migrate where their replacement or bystander effect is needed. Indeed, endogenous NSCs migrate out of the niche at physiological rate to maintain brain homeostasis, either differentiating or releasing tropic factors (Shen et al., 2004; Kokaia et al., 2012). When transplanted during acute or chronic neuroinflammatory disorders, NSCs show remarkable pathotropism: they follow the molecular gradient of chemotactic inflammatory factors (Muller et al., 2006) and reach the damaged site where they start secreting a series of molecules (i.e., bone morphogenetic protein 4, noggin, Notch, Jagged, and SHH) to recapitulate the microenvironment of the SVZ niche (atypical ectopic perivascular niche) (Pluchino et al., 2003, 2010; Irvin et al., 2004; Stidworthy et al., 2004; Martino and Pluchino, 2006; Bonaguidi et al., 2008).

Of note, beside communication *via* soluble factors, NSCs sense and can release intracellular messengers wrapped in vesicles (Cossetti et al., 2014). Although their role in adult NSCs is almost unexplored (more is known for other types of stem cells), vesicles can transfer information in the form of mRNA, ribosomal RNA, long non-coding RNA, microRNA, DNA, protein, or lipids (Thery et al., 2002; Huang et al., 2013; Batiz et al., 2015; Kirby et al., 2015).

## NSC function in neuroinflammation and neurodegeneration. focus on multiple sclerosis and ischemic stroke.

The peculiar plasticity of the CNS and of its NSCs manifests after CNS injury, when (i) proliferation and differentiation of NSCs is enhanced; (ii) striatal spiny interneurons and glutamatergic neurons are ectopically found in the injured cortex and in the striatum after stroke (Thored et al., 2007) while migrating neuroblasts become oligodendrocytes in area of demyelination after exiting the niche; (iii) ependymal cells behave as progenitors (Luo et al., 2008) and directly convert into neurons (Carlen et al., 2009); (iv) reactive astrocytes in ischemic brain injury exhibit self renewal capacity and multipotency (Buffo et al., 2008; Gabel et al., 2016).

The fact that non-neurogenic precursors can convert into neurogenic cells clearly highlights how exogenous factors can trigger plasticity within and outside of the niche (Carlen et al., 2009; Magnusson et al., 2014; Shetty and Hattiangady, 2016). Moreover, during pathology and steady-state, NSCs also exert trophic non-neurogenic functions which are crucial to maintain brain homeostasis.

In the following section we will explore how NSC niches function during CNS injury, with focus on stroke, multiple sclerosis and their animal models (Figures 1B,C, Table 1).

### Effect of oxygen supply on neural stem cell plasticity, connection with stroke, and multiple sclerosis

In physiological conditions, neural stem cells are exposed to an oxygen concentration between 2.5 and 5.0%, which promotes NSC self-renewal *via* VEGF and erythropoietin (EPO) production induced by hypoxia inducible factor 1α, HIF1α (Pavlica et al., 2012; Li et al., 2014).

In the quiescent state, NSC mitochondria are quite immature with globular shape, do not depend much for energy on oxidative phosphorylation (OXPHOS), rather on glycolysis, with high lactate production (Zheng et al., 2016). Although glycolysis produces less ATP than mitochondrial OXPHOS, the pathway is very fast in NSCs (Ito and Suda, 2014). Their anaerobic metabolism is sustained mainly by mitochondria uncoupling protein 2 (UCP2), high level of hexokinase II and low pyruvate dehydrogenase to keep under control the production of reactive oxygen species (ROS) (Madhavan et al., 2006; Zheng et al., 2016). In this way, DNA and proteins of the cells are protected from ROS-dependent potential damage. Further, ROS, produced in limited amount in physiological conditions and normally neutralized, are also beneficial because they trigger self-renewal and neurogenesis (Le Belle et al., 2011). Conversely, differentiated cells present elongated, crystae-rich mitochondria, higher ratio of mitochondrial glucose oxidation (OXPHOS) over glycolysis as metabolic support (Zhang et al., 2014; Marcialis et al., 2016). Moreover, NSCs increase Krebs' cycle functionality and decrease lactate production, concurrent with increased number and total mitochondrial mass (Sola et al., 2013).

On the other side, the first consequence of the blood flow occlusion in brain ischemia is hypoxic injury that causes extensive neural cell death (Niquet et al., 2003). Nonetheless and surprisingly, hypoxia, while being detrimental in the acute phase of stroke, in a second step, contributes to promote neurogenesis (Arvidsson et al., 2002; Tonchev et al., 2003; Jin et al., 2006; Macas et al., 2006; Minger et al., 2007; Wang et al., 2011; Zhang and Chopp, 2016), to support replenishment of neurons in the RMS, migration in the region of ischemic brain injury, and growth of oligodendrocyte progenitors that disperse to the gray and white matter (Zhang et al., 2001, 2012; Parent et al., 2002; Minger et al., 2007; Li et al., 2010a; Zhang and Chopp, 2016).

Hypoxia has recently emerged as a concurrent complication of disease progression also in MS. Indeed, important lack of oxygen, occurring as a consequence of inflammation, has been measured in brain gray matter regions of MS patients, where it can *per se* be considered co-causative for neurodegeneration (Haider et al., 2014; Yang et al., 2015). Since hypoxia and inflammation are strictly linked, it is still difficult to tease apart their respective contributions in MS (Sun et al., 1998; Lassmann, 2003; Davies et al., 2013). It looks like hypoxia can occur in MS patients as a consequence of increased oxygen demand. When this request is not satisfied, hypoxia can be further detrimental for surrounding neural stem cells (Trapp and Stys, 2009).

### Effect of inflammation on neural stem cell plasticity, connection with stroke, and multiple sclerosis

It has been well-documented that the brain cannot be simplistically considered an immune privileged site (Kleine and Benes, 2006) and actually immune system activation in the brain exerts both damaging and beneficial effects on CNS functions (Martino, 2004), depending on the onset of the inflammation, on the cell types involved in the process and on the chronicity of the response (Crutcher et al., 2006; Kyritsis et al., 2012).

NSCs share with the immune system an array of secreted mediators and receptors, which are all relevant for the maintenance of the neurogenic niche and, at the same time, represent the prerequisite for the interaction of NPCs with the microenvironment, especially during neuroinflammation (De Feo et al., 2012; Kokaia et al., 2012).

Indeed, inflammation, *via* its associated cues (i.e. cytokines, chemokines, chemical species….), strongly impacts structure and function of the stem cell niche, acts directly on NSCs and affects tissue restoration/regeneration *via* microglia and astrocyte activation (Ekdahl et al., 2003; Pluchino et al., 2008; Pourabdolhossein et al., 2017).

In homeostatic conditions, microglia are ramified in shape to maintain surveillance. Upon pathogen invasion or insults, they retract the protrusions, become amoeboid, increase their migratory behavior, and secrete cytokines. IL6, TNFα, IL1β, and complement 1 subunit q (C1q) are among the most potent microglia–derived cytokines, able to compromise the niche environment, inhibit neurogenesis (Vallieres et al., 2002; Monje et al., 2003), induce oligodendrogenesis (Valerio et al., 2002) and a subtype of reactive astrocytes (Liddelow et al., 2017). Further, while NSC-derived nitric oxide synthase promotes release of small amounts of nitric oxide (NO) with neurogenic properties (Carreira et al., 2010; Luo et al., 2010), abundant NO released by microglia or astrocytes in inflammatory conditions (i) inhibits proliferation of NSCs acting on the EGF receptor (Carreira et al., 2014) or on the transcription factor complex Neuron-Restrictive Silencer Factor /RE1-Silencing Transcription factor, NRSF/REST (Bergsland et al., 2014) and (ii) promotes gliogenesis (Bergsland et al., 2014).

IFNγ, a pleiotropic cytokine, has a central role in regulating NSCs proliferation and quiescence (Kulkarni et al., 2016). In inflammatory conditions, such as experimental autoimmune encephalomyelitis (EAE), the preclinical model of MS, IFNγ dramatically decreases progenitor proliferation (Pluchino et al., 2008; Pereira et al., 2015) and inhibits the recruitment of newborn neurons to the olfactory bulb (OB). IFNγ might also play a crucial role in regulating glioma-associated oncogene homolog-1 (Gli1), a Shh-induced trasncription factor that drives the oligodendroglial fate of NSCs (Wang et al., 2008; Li et al., 2010b; Samanta et al., 2015).

The complement system also inhibits NSC activity as demonstrated with complement receptor 2 (Cr2) knock out mice that have increased basal neurogenesis (Moriyama et al., 2011). Moreover, lipid modifiers present in inflammation, such as leukotriene, can negatively regulate NSC proliferation likely *via* TNFα and IL1β (Bonizzi et al., 1999; Wu et al., 2013). Of note, montelukast, a leukotriene receptor blocker protects from this event (Marschallinger et al., 2015).

IL6 is another crucial mediator of NSC function and its induction, e.g., during maternal infection and stroke, permanently perturbs NSC proliferation and neurogenesis (Gallagher et al., 2013; Chucair-Elliott et al., 2014; Meng et al., 2015).

Conversely, the anti-inflammatory interleukin-10 (IL10) in rodents keeps NSCs in an undifferentiated proliferation state, rather than promoting differentiation (Ben-Hur et al., 2003; Perez-Asensio et al., 2013; Wang et al., 2015).

Nevertheless, as anticipated, inflammation can be also beneficial for NSCs. First, the immune system in general and T cells in particular are involved in maintaining niche neurogenesis, as indeed genetic T-cell depleted mice present compromised cognitive functions (Ziv et al., 2006). Moreover, chemokines such as monocyte chemoattractant protein 1 (MCP1) or SDF1, released in inflammatory conditions, promote migration of NSCs to site of injury and local TNFα or IL1β trigger their proliferative capacity (Widera et al., 2006). Second, injection of moderately activated microglia can regulate brain homeostasis and neurogenesis (Butovsky et al., 2006; Bachstetter et al., 2011).

Further, NSCs directly produce inflammatory chemokines (Covacu et al., 2009), promoting a feed forward loop at sites of tissue damage (Imitola et al., 2004; Belmadani et al., 2006; Widera et al., 2006; Wang et al., 2007). NSCs can also directly change inflammatory responses through immunomodulatory factors (Pluchino et al., 2005; Ben-Hur, 2008; Yong and Rivest, 2009; Butti et al., 2012) or trophic factors (Huang et al., 2014). In particular, SVZ-NSCs can protect striatal neurons from the excitotoxic damage occurring in stroke and epilepsy, releasing endogenous endocannabinoids, likely upon inflammatory *stimuli* (Butti et al., 2012). Endogenous NSCs also secrete MCP1, VEGF, IL4, and interleukin-15 (IL15), the latter known to retain NK cells in the chronic phase of EAE in mice and of MS in humans. Indeed, in the SVZ, NK cells contribute to the dysfunction of the neurogenic niche observed in EAE by killing stem cells (Liu et al., 2016). Moreover, inflammatory cytokines lead to metabolic reprogramming of the arginase pathway in NSCs ultimately impacting on the NSC mediated anti-proliferative effect on T cells (Drago et al., 2016). Further, it has been reported that human NSCs hinder the differentiation of myeloid precursor cells into immature dendritic cells and the final maturation into functional antigen presenting cells (Pluchino et al., 2009).

In stroke, immune cell infiltration and inflammation take place as secondary events to hypoxic injury, which is responsible of the massive neuronal death. Upon brain injury, the blood-brain barrier (BBB) is disrupted, its permeability increases, migrating immature neurons associate with the angiogenic fenestrated endothelium, thus mimicking the neurogenic niche structure (Ohab et al., 2006; Ohab and Carmichael, 2008). Neutrophils and innate immune cells are the first players, although also T and B cells cross the damaged-BBB, inducing a rapid adaptive autoimmune response to neuronal antigens (Chamorro et al., 2012). As consequence of inflammation, neurogenesis is activated in stroke (Wang et al., 2011; Katajisto et al., 2015) supported by soluble factors such as bone morphogenic protein (Forni et al., 2013), retinoic acid (Plane et al., 2008), sonic hedgehog (Cheng et al., 2015), C-C motif chemokine Ligand 2 (CCL2) (Osman et al., 2016) along with SDF1 and angiopoietin-2 (Ang2) that home NSC progenitors to the site of injury (Thored et al., 2006), where they improve functional recovery (Guzman et al., 2008) and differentiate into neurons (Darsalia et al., 2007). Nevertheless, they present only limited reparative properties because matured neurons tend to die (Arvidsson et al., 2002; Marti-Fabregas et al., 2010; Kazanis et al., 2013). Moreover, given that NSCs themselves express and release respectively C-X-C motif chemokine receptor 4 (CXCR4) and SDF1, they might play a role in regulating axonal remodeling in ischemic brain. On the other hand, SVZ-derived astrocytes have a crucial role in ischemic stroke: photothrombotic cortical ischemia induces a strong gliogenic, Notch1-dependent response in the SVZ, which generates thrombospondin-4 (Thbs4)-positive astrocytes. Thbs4 astrocytes are essential components of the glial scar and their proliferation might be favored over neurogenesis after cortical injury (Benner et al., 2013). Conversely, it has also been reported that inflammation can promote neurogenesis in dormant neural progenitors in nonconventional neurogenic regions (Jiao and Chen, 2008).

Further, stroke increases generation of oligodendrocyte precursor cells (OPCs) from NSC in the ischemic brain, although it remains to be defined whether NSC-derived OPCs, beside oligodendrogenesis, help with brain repair, likely communicating with cerebrovasculature and other brain parenchyma cells (Itoh et al., 2015).

Overall, according to several evidences, inflammation in stroke can be beneficial for NSCs, nonetheless other reports, evaluating microglia function, claim that activated ED1^+^ microglia impair basal neurogenesis (Ekdahl et al., 2003) likely *via* TNFα/TNFR1 signaling (Iosif et al., 2008; Chen and Palmer, 2013; Gebara et al., 2013). In humans, a definitive robust evidence of clinical post-stroke neurogenesis remains a matter of investigation as analysis of autopsy tissue provided positive results (Ekonomou et al., 2012) which are instead lacking when labeling newly born neurons with ^14^C (Huttner et al., 2014).

In MS preclinical models, it is important to distinguish among experimental conditions since they influence the experimental evidence. Nonetheless, either chemically (LPC), focal (site injection of TNFα-IFNγ combined with subclinical immunization) or immune-mediated (EAE) demyelination causes reduced neuroblast proliferation in the olfactory bulb of mice, while neutralization of immune mediators, such as IFNγ and Galectin3 inflammatory cytokines, restores neurogenesis both *in vitro* (Pluchino et al., 2008; Tepavcevic et al., 2011) and *in vivo* (Monje et al., 2003; James et al., 2016). Indeed, in human samples, the SVZ of post-mortem MS brains is altered, with reduced number of neuroblasts in the SVZ of the lateral ventricle. Conversely, neuroinflammation increases neurotrophin levels (e.g., nerve growth factor-NGF) in the SVZ (Calza et al., 1998; Triaca et al., 2005), likely counteracting the inhibitory effect of proinflammatory molecules. Further, demyelination is able to boost proliferation of NSCs in the neurogenic niches promoting their differentiation (Pluchino et al., 2005; Menn et al., 2006). Only in conditions of acute cytokine exposure, either by focal intrathecal cytokine injection or as a consequence of strong microglia activation, the proliferative capacity of the niche is inhibited. In terms of gliogenic differentiation, inflammatory conditions may increase the percentage of Olig2^+^ cells originating from the SVZ *via* modulation of bone morphogenetic protein (BMP) signaling (Tepavcevic et al., 2011). The transcription factor Gli1 seems to be a key regulator of SVZ oligodendrogenesis and may be a promising target for reparative strategies in MS, as pharmacological inhibition of Gli1 during EAE promotes remyelination and improves clinical outcome (Samanta et al., 2015).

Leveraging the regenerative potential of NSCs and their immunomodulatory and trophic functions, NSC transplant has been proposed as therapeutic strategy for inflammatory diseases of the CNS. In stroke, transplanted NSCs directly modulate neuronal circuit plasticity (Zhang et al., 2009) through the expression of developmental molecules such as guidance molecules (i.e., slit, thrombospondin-1 and -2) and trophic factor such as VEGF (Andres et al., 2011). In fact, transplanted NSCs in stroke stimulate the proliferation of endogenous neural stem cells (Zhang et al., 2011; Hassani et al., 2012; Mine et al., 2013), increase endogenous angiogenesis after stroke (Jiang et al., 2005), scavenge the neurotoxic molecules (Emsley et al., 2004) and contribute both directly and indirectly (*via* astrocytes) to glutamate clearance (Bacigaluppi et al., 2009, 2016).

In EAE, transplanted NSCs directly inhibit both T cell and myeloid cell immune responses. In the CNS, they block inflammatory cell recruitment, T cell proliferation and promote the apoptosis of brain-reactive T cells (Einstein et al., 2003; Pluchino et al., 2005). In peripheral secondary lymphoid organs of EAE mice, antigen-specific proliferation of T cells, dendritic cell antigen presentation and chemotactic gradients are impaired by NSCs transplanted either subcutaneously or intravenously. As an example, NSC-secreted LIF, on one hand, stimulates endogenous remyelination in the CNS (Laterza et al., 2013), on the other, inhibits Th17 cell differentiation in the periphery (Cao et al., 2011).

Overall, the current literature shows that a controlled use of inflammation in CNS injury could be of help in regenerative approaches when NSC proliferation needs to be boosted and inflammatory tweaking can have beneficial outcomes. An option could consist in reducing NSC sensitivity to inflammatory mediators and, at the same time, increasing the therapeutic efficacy of NSCs when transplanting engineered cells.

### Effect of aging on neural stem cell plasticity, connection with stroke, and multiple sclerosis

Aging is a common feature of both stroke and progressive multiple sclerosis and loss of niche integrity, depletion of the stem cell pool, cellular senescence, defect in cell-cell contact in the niche or metabolic changes are all shared characteristics that contribute to the demise of the aging neurogenic niche (Oh et al., 2014).

The most significant consequence of age in the SVZ consists in the alteration of the niche cytostructure and in reduction of the neuroblast population, with reduced proliferation and neurogenic capacity (Kerever et al., 2015). On the contrary, oligodendrogenesis is substantially not affected by aging, rather oligodendrocyte recruitment and differentiation are impaired (Sim et al., 2002; Franklin and Ffrench-Constant, 2008; Conover and Todd, 2016). The latter is indeed a causal pathogenic characteristic of chronically demyelinated MS lesions in humans (Kuhlmann et al., 2008).

Concurrently, given that senescence does not spare the vasculature (Farkas and Luiten, 2001), the environment of the niche may enrich for toxic factors which further sustain exhaustion, reduce neuroplasticity and cause cognitive decline (Villeda et al., 2011). Potential therapeutic strategies to counteract the decline of adult stem cells may involve the promotion of a rejuvenating environment (Katsimpardi et al., 2014) or the prevention of premature exhaustion of long-lived self-renewing NSC populations. Neurogenic decline in aged SVZ can occur because of the accumulation of damaged DNA and both nuclear and mitochondrial DNA are susceptible to age-related changes (Bailey et al., 2004). Upon sensing DNA damage, SVZ cells upregulate the inhibitor of cyclin kinase to post-pone cell cycle entry and reduce proliferation of NSCs (Molofsky et al., 2006).

NSC age-dependent cell plasticity is also influenced by metabolic changes (Rabie et al., 2011; Chaker et al., 2015). As far as nutrient sensing pathways, growth differentiation factor-11 (GDF11), has been identified as one of the extrinsic circulating youth signals that maintain neurogenesis (Katsimpardi et al., 2014), although those findings are currently matter of further validation (Egerman et al., 2015; Smith S. C. et al., 2015). It is still unknown whether the effect is direct onto NSCs or indirect *via* improved microvascular network. IGF1, another critical growth factor, *per se* stimulates proliferation and differentiation of NSCs (Aberg, 2010), but decreases with aging (Sonntag et al., 2005). Nonetheless, it is also life–long exposure to IGF1 that with age causes decline of neurogenesis (Chaker et al., 2015). Along the same line, NAD^+^ and AMP levels, which reflect cellular energy state, decline with age in the hippocampus, in parallel with reduction of differentiation and self-renewal capacity of NSCs, because their regulatory enzyme decreases (Liu et al., 2012; Stein and Imai, 2014). As far as oxygen-dependent effect, those are linked to failure in maintaining appropriate mitochondrial regulations. Indeed, accumulation of mutations in mitochondrial DNA (mtDNA) occurs during aging and comes along with abnormal accumulation of toxic by-products (Siqueira et al., 2005) because mtDNA lacks protective histones and is located close to the major source of ROS, the electron transport chain (Park and Larsson, 2011).

Inflammation is another comorbidity factor associated with aging. The immune molecules C-C motif chemokine-11 (CCL11) and β2-microglobulin (B2M), as well as CCL2, C-C motif chemokine-19 (CCL19) and haptoglobin, contribute to low-grade of inflammation in aging (inflammaging) (Franceschi and Campisi, 2014), and negatively affect neurogenesis (Villeda et al., 2011; Wyss-Coray, 2016). Indeed, heterochronic parabiosis between aged and young mice rejuvenates and reverses stem cell aging in numerous tissues (Conboy et al., 2005) or administration of plasma from young mice can ameliorate cognitive impairment in aged mice (Villeda et al., 2014).

Last, it is worth mentioning that also the cellular environment of the niche sustains aging features. Astrocytes of the niche, with age, reduce release of Wnt3 (Lie et al., 2005; Okamoto et al., 2011), that in turn down regulates Survivin expression and increases release of Dickkopf-related protein 1 (DKK1), a canonical Wnt signaling inhibitor. The final effect is induced quiescence on one side (Miranda et al., 2012) and negative regulation of NSC neurogenesis (Seib et al., 2013) on the other. This is paired with physical changes of the extracellular matrix, which also affect senescence of the niche (Gattazzo et al., 2014). Conversely, it is also possible that NSCs efficiently graft the SVZ and well differentiate in both young and aged hippocampus, suggesting that advanced age of the host at the time of grafting has no major adverse effects on engraftment, migration, and differentiation of SVZ-NSCs (Shetty and Hattiangady, 2016).

Age is an independent risk factor for brain ischemia (Marinigh et al., 2010; Roger et al., 2012) and stroke models using aged animals are clinically very relevant (Popa-Wagner et al., 2007). From work on animals, it has been reported a 50% decline in neurogenesis in the SVZ of elder compared with young–adult animals (Darsalia et al., 2005; Macas et al., 2006; Ahlenius et al., 2009). Unfortunately, although endogenous neurogenesis has been observed even in aged humans, it is still not clear to what extent newly formed neurons are functionally relevant for stroke recovery in human patients. Thus, cell therapies have been implemented to address this question. An open-label, single-site, dose-escalation study was performed on 13 aged patients after ischemic stroke, transplanting by stereotaxis the neural stem cell line CTX0E03 in the ipsilateral putamen. After 2 years, improvement in the disability score scale (NIHSS) ranging from 0 to 5 (median 2) points has been recorded (Borlongan, 2016; Kalladka et al., 2016). Similarly, studies in animal models examined whether exogenous NSC delivery might restore neurogenesis in the DG of old rodents. Both embryonic and fetal derived NSCs stimulated neurogenesis in young or old animal models. Likely the effect was exerted *via* suppression of microglia/macrophage activation (Jin et al., 2011). Of note, inhibition of microglial activation has also been reported in ischemic mice following systemic delivery of mouse NSCs (Bacigaluppi et al., 2009).

Those data suggest that the problem with NSCs in ischemic stroke is to some extent cell autonomous, because the environment of the aged brain does not preclude NSC engraftment.

In MS, loss of myelin, a specialized membrane produced by oligodendrocytes, essential for normal CNS function, is a characteristic feature. Aging impairs not only the neurogenic but also the oligodendrogenic properties of the brain germinal niches as a consequence of longer cell cycle length of NSCs and of their progeny, loss of growth factors and upregulation of inhibitors (Hamilton et al., 2013). Iron accumulation (Andersen et al., 2014), linked in cascade to oxidative alteration and to mitochondrial dysfunction (Mahad et al., 2015) are other recognized characteristics of neurodegeneration in progressive aged MS individuals. A direct impact also on neural stem cells has not been experimentally proven (Crabbe et al., 2010) but can reasonable be speculated.

In the context of demyelination, work by Crawford et al. demonstrated responsiveness of OPCs to demyelination, although remyelination and susceptibility to aging is regionally dependent: in chemically-induced demyelination of the corpus callosum in aged animals, dorsal SVZ-NSCs are efficiently recruited to lesions and their differentiation into OPCs is impaired, whereas ventral NSCs are recruited more slowly but differentiate rapidly into OPCs; similarly, while in aged animals the percentage of differentiated OPCs from dorsal NSCs is half of the one seen in young animals, the percentage of ventral NSCs differentiating into OPCs remained constant (Crawford et al., 2016). This evidence has significant implications for the progressive form of MS, for which age is the current best associated risk factor (Minden et al., 2004; Scalfari et al., 2011). As disease progresses with age, repair, remyelination and other physiological functions become less robust. Animal models have shown that while OPC recruitment toward lesions remains intact, differentiation into myelinating oligodendrocytes decreases with age with a slower and less effective remyelination process compared to young rats (Rist and Franklin, 2008; Ruckh et al., 2012). Similarly, elder human subjects showed reduced remyelinating capabilities. Multiple hypotheses can explain these findings, including either extrinsic factors or intrinsic characteristics such as epigenetic changes known to be modulated during aging and thus to impact OPC maturation (Rist and Franklin, 2008).

## How do neural precursors from induced pluripotent stem cells stand?

The technology of somatic cell reprogramming (induced pluripotent stem cells, iPSCs) that developed in the last 10 years (Takahashi et al., 2007), represents an invaluable alternative strategy to obtain, in large scale and indefinitely, neural precursors, unfolding new research and clinical avenues. Expandable intermediate neural precursors can be obtained with published protocols (Falk et al., 2012; Reinhardt et al., 2013) and the cells resemble most of the specific properties of endogenous plastic cells. To treat neurodegenerative conditions, both iPSC-derived NSCs or terminally differentiated neurons have been transplanted in preclinical models. Early on, it has been demonstrated that those cells are able to survive and adequately integrate into the host brain (Oki et al., 2012; Tornero et al., 2013; Qi et al., 2017), and recently it was reported that transplanted human iPSC-derived cortical neurons can be incorporated also into injured cortical circuits and could contribute to functional recovery (Tornero et al., 2017). Beside reparative activity, transplanted cells can improve the brain functional outcome, directly waking up brain plasticity *via* their bystander effects. Indeed, transplanted cells promote the formation of new synapses among existing neurons or preserve axonal integrity releasing trophic factor for myelinating cells as described in ischemic stroke and in a preclinical model of MS (Oki et al., 2012; Espuny-Camacho et al., 2013; Laterza et al., 2013; Tornero et al., 2013).

In addition, the release of soluble factors by (neural) stem cells along with cell-cell interactions supports angiogenesis that, beside self organizing blood vessels (Kusuma et al., 2013), guarantees trophic support for proper integration of neuronal cells and sustains synaptogenesis. Further, the bystander effect is exploited also in terms of (i) inhibition of neutrophil and peripheral dendritic cell activation and recruitment, (ii) inhibition of effector T- and B-cells, (iii) attenuation of BBB damage, or (iv) stimulation of the M2 microglial phenotype (Drago et al., 2013). Similar evidence of the (neuro)-protective, regenerative, and angiogenic properties of iPSCs is not available yet from their complete secretome, nonetheless it can be reasonably predicted.

As of now, while work by Jensen et al. reported no iPSC-mediated improvement in behavioral function in stroke (Jensen et al., 2013), several others, as previously mentioned, show that also direct iPSC transplantation improves neurological score, motor, sensory and cognitive functions, already within the first week after transplantation, when migration, maturation, synaptic integration, and paracrine release into the host brain is completed (Oki et al., 2012; Polentes et al., 2012; Tornero et al., 2013, 2017; Yuan et al., 2013; Tatarishvili et al., 2014).

Similarly, transplant of iPSC-derived neural precursors in the MS context dramatically reduced T cell infiltration and ameliorated white matter damage in treated EAE mice (Laterza et al., 2013; Zhang C. et al., 2016), both *via* improved differentiation toward cells of the oligo-glial lineage and *via* soluble factor release (Laterza et al., 2013).

Experimental validation in humans, both for stroke and MS, could be useful in the future but regulatory guidelines for safe clinical trial are awaited.

## Can we guide neural stem cell plasticity toward a desired response?

Taking advantage of their plasticity, stem cells can generate, repair, and change nervous system functions. Fine-tuning of the inflammatory responses or proper modulation of the hypoxic and aging conditions should provide a permissive milieu for an effective reparative process. In this sense, the perspective that aged hippocampus licenses efficient and functional engraftment (Shetty and Hattiangady, 2016) puts forward the possibility that grafted niches can continuously generate new neurons and glia which, in turn, are expected to improve the plasticity and function of aged brain tissue.

As engineering and reprogramming approaches advance, transplant of exogenous human NSCs are optimized and better understanding of NSC regulation in physio- pathological conditions is achieved, it will be possible to fully take advantage of the therapeutic potential of those cells and successfully exploit their plasticity.

So far, it is possible to (i) engineer NSCs to deliver extrinsic stem cell fate determinants or intrinsic factors that allow the development of biomimetic environmental cues to control stem cell fates (Michailidou et al., 2014; Karimi et al., 2016); (ii) culture and expand stem cells outside of their native environments (Komura et al., 2015); (iii) perform localized delivery of microspheres to specifically differentiate endogenous stem cells (Gomez et al., 2015); (iv) engineer somatic cells to obtain induced NSCs with reparative functions (Hargus et al., 2014); (v) engineer human stem cells to obtain indefinitely expandable lines for clinical purposes (Pollock et al., 2006); (vi) snapshot the metabolic profile of cells in physio-pathological conditions (Bystrom et al., 2014; Dislich et al., 2015).

Systems biology will likely represent a fundamental tool to discover novel drugs to modulate NSC plasticity, as it can integrate and analyze in a quantitative manner several aspects of NSC behavior (e.g., differentiation potential and self-renewal) (Wells et al., 2013; Hussein et al., 2014).

## Conclusions and future outlook

Neural progenitor cells represent an underdeveloped therapeutic resource in clinical settings. Although their paracrine and endocrine bystander potential is well-known, much of their plasticity still needs to be properly manipulated for therapeutic purposes.

On one end, cells can be expanded and bioengineering approaches are becoming available to further potentiate their availability (Bressan et al., 2014; Bouzid et al., 2016; Gardin et al., 2016). For instance, *in vitro* preconditioning with low oxygen tension might enhance NSC survival as it mimics the oxygen supply normally found in brain tissues (Hawkins et al., 2013; Sandvig et al., 2017). Nonetheless, it is this same plasticity that could threaten their therapeutic capacity, because the manipulation of cells themselves or of the trophic microenvironment, might induce unwanted side-effects, such as senescence from over-stimulation. Thus, it might be worth increasing the knowledge on (i) the influence of cell metabolism on NSC behavior during CNS diseases; (ii) the *ex-vivo* maintenance of NSCs; (iii) the most suitable window time for transplant to best leverage graft survival and disease specific environmental conditions. Indeed engraftment efficiency strongly correlates with therapeutic benefits.

Furthermore, this additional knowledge should shed light on the age-related decline of the stem cell niche, which contributes to neurodegeneration in both chronic and acute diseases. Last, careful analysis of the microenvironment is warranted to overcome complications and to improve the efficacy of cell therapy approaches.

## Author contributions

LO, AM wrote and approved the manuscript. GM, developed the concept wrote and approved the manuscript.

### Conflict of interest statement

The authors declare that the research was conducted in the absence of any commercial or financial relationships that could be construed as a potential conflict of interest.
